# ZBTB20 in Nociceptive Neurons of the Trigeminal Ganglia Regulates Pruritus

**DOI:** 10.3389/fmed.2021.626554

**Published:** 2021-03-04

**Authors:** Xin Jia, Meng-Han Dai, An-Jing Ren, Ting-Ting Wang, Weiping J. Zhang, Ling Zhang

**Affiliations:** ^1^The First Rehabilitation Hospital of Shanghai, Tongji University School of Medicine, Shanghai, China; ^2^Key Laboratory of Spine and Spinal Cord Injury Repair and Regeneration of Ministry of Education, Orthopaedic Department of Tongji Hospital, School of Medicine, Tongji University, Shanghai, China; ^3^Department of Anesthesiology, Renmin Hospital, Hubei University of Medicine, Shiyan, China; ^4^Department of Pathophysiology, Naval Medical University, Shanghai, China; ^5^Department of Dermatology, Tongren Hospital Shanghai Jiao Tong University School of Medicine, Shanghai, China; ^6^NHC Key Laboratory of Hormones and Development, Tianjin Institute of Endocrinology, Tianjin Medical University Chu Hsien-I Memorial Hospital, Tianjin, China

**Keywords:** itch, TRPA1, TRPV1, ZBTB20, trigeminal ganglia, pain, pruritus

## Abstract

Recent studies have shown that ZBTB20, a zinc-finger protein containing transcription factor, is highly expressed in small-diameter primary sensory neurons in mice, and modulates pain through regulating TRP channels. However, whether ZBTB20 regulates itch sensation has not been demonstrated. In this study, small-diameter primary sensory neuron-specific ZBTB20 knockout (PN-ZB20KO) mice were used to investigate the role of ZBTB20 in the regulation of itch sensation. First, both histamine-dependent and non-histamine-dependent itch behaviors induced by injection of histamine and chloroquine (CQ) into the cheek were significantly diminished in PN-ZB20KO mice. Second, double immunohistochemistry showed that ZBTB20 was mainly expressed in CGRP-labeled small peptidergic neurons and was expressed at low levels in IB4-labeled small non-peptidergic and NF200-labeled large neurons in the trigeminal ganglia (TG). ZBTB20 was also expressed in most TRPV1^+^ and TRPA1^+^ neurons and to a lesser extent in TRPM8^+^ neurons in the TG. Furthermore, cheek injection of histamine and CQ enhanced the mRNA expression of TRPV1 and TRPA1 but not TRPM8 in the TG. Moreover, TRPV1 and TRPA1 knockout (KO) mice exhibited attenuation of itch behavior induced by histamine and CQ, respectively. Finally, silencing endogenous ZBTB20 with recombinant lentivirus expressing a short hairpin RNA against ZBTB20 (LV-shZBTB20) in TG neurons attenuated histamine- and non-histamine-induced itch and downregulated TRP channels in the TG. Our study suggests that ZBTB20 plays an important role in mediating itch in small primary sensory neurons.

## Introduction

The zinc finger protein ZBTB20 regulates development and metabolism in multiple systems and is essential for postnatal survival in mice (1). ZBTB20 has been found to play a crucial role in the development and function of the central nervous system, such as the development of dendritic and synaptic structures (2), the maturation of CA1 neurons (3), and the generation of neuronal layers in the developing cortex (2, 4). However, the function of ZBTB20 in the peripheral nervous system has not been fully investigated.

Recently, ZBTB20 was specifically knocked out in nociceptive neurons in mice, alerting the expression of transient potential (TRP) channels, including TRPV1, TRPA1, and TRPM8, and thus resulting in abnormal mechanical pain, heat pain and inflammatory pain (5). TRP channels are a large family composed of 28 members in mammals that can be divided into seven subfamilies, including TRPA, TRPC, TRPM, TRPN, TRPML, TRPP, and TRPV, based on their amino acid sequence homology (6, 7). Many TRP channels have been found to participate in the transduction of thermal, chemical, and mechanical sensations (8, 9). TRPV1, TRPA1, and TRPM8, which have long been reported to play important roles in the transduction of a variety of noxious stimuli (10, 11), have recently been implicated in the processing of itch sensation (12, 13).

Itch, an unpleasant sensation that provokes the scratch reflex (14), can be classified as histamine-dependent and non-histamine-dependent according to the sensitivity of the sensation to antihistamine treatment (15). Histamine and non-histamine itch have been reported to be mediated by distinct TRP signaling pathways (16–18). Although itch and pain are both mediated by primary sensory neurons, the cell bodies of which are located in the dorsal root ganglia (DRG) or trigeminal ganglia (TG) (14), they are distinguished by unique behavioral responses (19). While pain evokes acute withdrawal behaviors to escape from nociceptive stimuli, itch leads to a scratch reflex and brings attention to the affected area to remove pruritogens and provides temporary relief. Itch was previously thought to be a kind of minor pain and not an independent sensory modality. Recently, progress has been made toward elucidating the molecular mechanism underlying itch. Itch and pain are now clearly understood to be distinct sensory modalities involving distinct neural and molecular pathways in primary sensory neurons and the spinal cord (20–25). Given the similarities and differences between pain and itch sensation, it is worth investigating whether ZBTB20 in primary sensory neurons regulates itch.

In the present study, we used PN-ZB20KO mice and gene silencing of ZBTB20 in the TG to specifically detect whether ZBTB20 in primary sensory neurons mediates itch sensation. We found that ZBTB20 was involved in both histamine- and non-histamine-dependent itch, and the effect was likely mediated by TRPA1 and TRPV1 channels in TG neurons.

## Materials and Methods

### Animals

ZBTB20 mutant (ZBTB20^flox/flox^; Nav1.8-Cre) mice, named PN-ZB20KO mice, were described previously (5). Floxed/Cre-negative, non-floxed Cre-positive, or wild-type (WT) mice were used as littermate controls. C57BL/6 mice were purchased from SLAC Laboratory Animal Company (Shanghai, China). All mice, including TRPV1 knockout (KO) and TRPA1 KO mice and their littermates, were provided food and water *ad libitum* and housed under a 12-h/12-h light/dark cycle. The temperature in the animal facility was maintained at 22 ± 1°C, and the relative humidity was 40–60%. The mice were allowed to adapt to the environment for 1 week before the experiment was initiated. Animal care procedures and experimental protocols were reviewed and approved by the Animal Study Committee of Tongji University School of Medicine (Shanghai, China).

### Drug and Administration

Pruritogens and algogen (histamine, H7125; chloroquine (CQ), C6628; capsaicin, M2028) were purchased from Sigma-Aldrich (St. Louis, MO, USA). To induce itch responses in the facial region, histamine (50 μg) and chloroquine (40 μg) were dissolved in 10 μL of PBS, and capsaicin (10 μg) dissolved in 10 μL of solution (7% Tween 80:20% ethanol:73% PBS) was intradermally injected into the cheek region as reported previously (26–28).

### Behavioral Test

The mouse cheek model was established to distinguish pain and itch behaviors (19). Itch and pain responses were evaluated as described previously (26, 27). The right cheek of each mouse was shaved 2 days before the behavioral experiment, and histamine, CQ and capsaicin were intradermally injected into the right cheek. A video camera (SONY HDR-Cx240) was positioned above the mice to record their behavior, and the numbers of ipsilateral forelimb wipes and hindlimb scratch bouts in the injection site in 5-min intervals over a 30-min period were determined in a blinded manner.

### Real-Time Quantitative RT-PCR

The mRNA levels of ZBTB20, TRPA1, TRPV1, and TRPM8 were analyzed by RT-PCR. The mice were decapitated, and the bilateral TGs were collected with sterilized instruments 30 min after histamine and CQ administration into bilateral cheeks. Total RNA was extracted with an RNA Extraction Kit (Takara). Isolated RNA was reverse-transcribed to synthesize first strand cDNA using a cDNA synthesis kit (Tiangen). The ABI 7500 Real-Time PCR System and SYBR Green I (Tiangen) were used for PCR. Real-time PCR mixtures were prepared, and the reaction conditions were set following the kit instructions. GAPDH was served as an internal control. The melting curve was used to evaluate the reliability of the PCR results. The threshold cycle (CT) value (the inflection point of the amplification curve) was determined, and the relative expression of target genes was calculated using the 2^−ΔΔCt^ method. The primer sequences for ZBTB20, TRPV1, TRPA1, TRPM8, and GAPDH are shown in Table 1.

**Table 1:** Primers sequence for RT-PCR.

**Primer**	**Forward**	**Reverse**
ZBTB20	CGGCGAGCGCTCCCTCTACAGTG	GCTTGCGGCAGTGCGTGGTCT
TRPV1	GGGAGGCCTGGCTTCTACTTTG	TGCCGGCACTCTGGTTCGT
TRPA1	GGCAATGTGGAGCAATAGCG	CAATAAGCTGCCCAAAGGTC
TRPM8	ACATCCCCTTCCCCTTCGTT	TCGCCAGCCTTACTTGATGTTATT
GAPDH	CCAATGTGTCCGTCGTGGATC	GTTGAAGTCGCAGGAGACAAC

### Immunohistochemistry

Mice were anesthetized with 10% chloral hydrate and perfused through the ascending aorta with PBS followed by 4% paraformaldehyde in 0.1 M phosphate buffer (pH 7.4). After perfusion, the TGs were removed and post-fixed with 4% paraformaldehyde for 4 h. The samples were cut into 14-μm-thick frozen sections on a cryostat. The sections were incubated with primary antibodies (mouse anti-TRPV1, 1:1,000, Abcam; rabbit anti-TRPA1, 1:500, Abcam; rabbit anti-TRPM8, 1:500, Abcam; mouse anti-CGRP, 1:1,000, Abcam; mouse anti-NF200, 1:1,000, Abcam; mouse anti-IB4-FITC, 1:1,000, Sigma; rabbit anti-ZBTB20, 1:1,000, Atlas Antibodies AB; rat anti-ZBTB20, 1:2,000, Abcam) overnight at 4°C, followed by secondary antibodies (Alexa Fluor 555 donkey anti-rabbit IgG, 1:1,000 and Alexa Fluor 488-conjugated donkey anti-mouse IgG 1:1,000, Invitrogen) at room temperature for 2 h. The sections were then observed under an epifluorescence microscope. All images were made into figures using Adobe Photoshop (Adobe Systems Incorporated, San Jose, CA), with only minor adjustments to the contrast and brightness settings if necessary.

### RNA Inference and TG Stereotaxic Injection

The recombinant lentivirus that expressed a short hairpin RNA (LV-shRNA) against ZBTB20 (shZBTB20) was used to silence endogenous ZBTB20 (29). Mice were anesthetized with 1% sodium pentobarbital (100 mg/kg, i.p.) and then placed in a stereotaxic apparatus. The skull of mice was exposed through the midline incision of scalp, and the microinjection glass pipette was inserted into TG through bilateral craniotomy with a hand-held drill (relative to bregma: anteroposterior (AP), −0.5 mm; mediolateral (ML), ±2.2 mm; dorsoventral (DV), −5.8 mm according to the mouse atlas of Paxinos and Watson). LV-shZBTB20 or scrambled shRNA (2.0 × 10^8^ TU/mL, 300 nL in volume) was microinjected into the bilateral TG at a rate of 25 nL per min with glass micropipettes. After a week of recovery, the mice were injected with histamine, CQ and capsaicin intradermally into the cheek for the behavior test. The bilateral TGs were collected for the measurement of the expression of ZBTB20 and TRP channels and immunohistochemistry study.

### Statistical Analyses

All data are expressed as the mean ± SEM. Statistical analyses were performed with GraphPad 7.0. Differences between groups were compared using 2-tailed Student's *t*-test. The time course data were analyzed by two-way ANOVA followed by a test of homogeneity of variance. The criterion for statistical significance was *p* < 0.05.

## Results

### ZBTB20 in Primary Sensory Neurons Is Involved in the Modulation of Histamine-Dependent and Non-histamine-Dependent Itch

**Figure 1:**
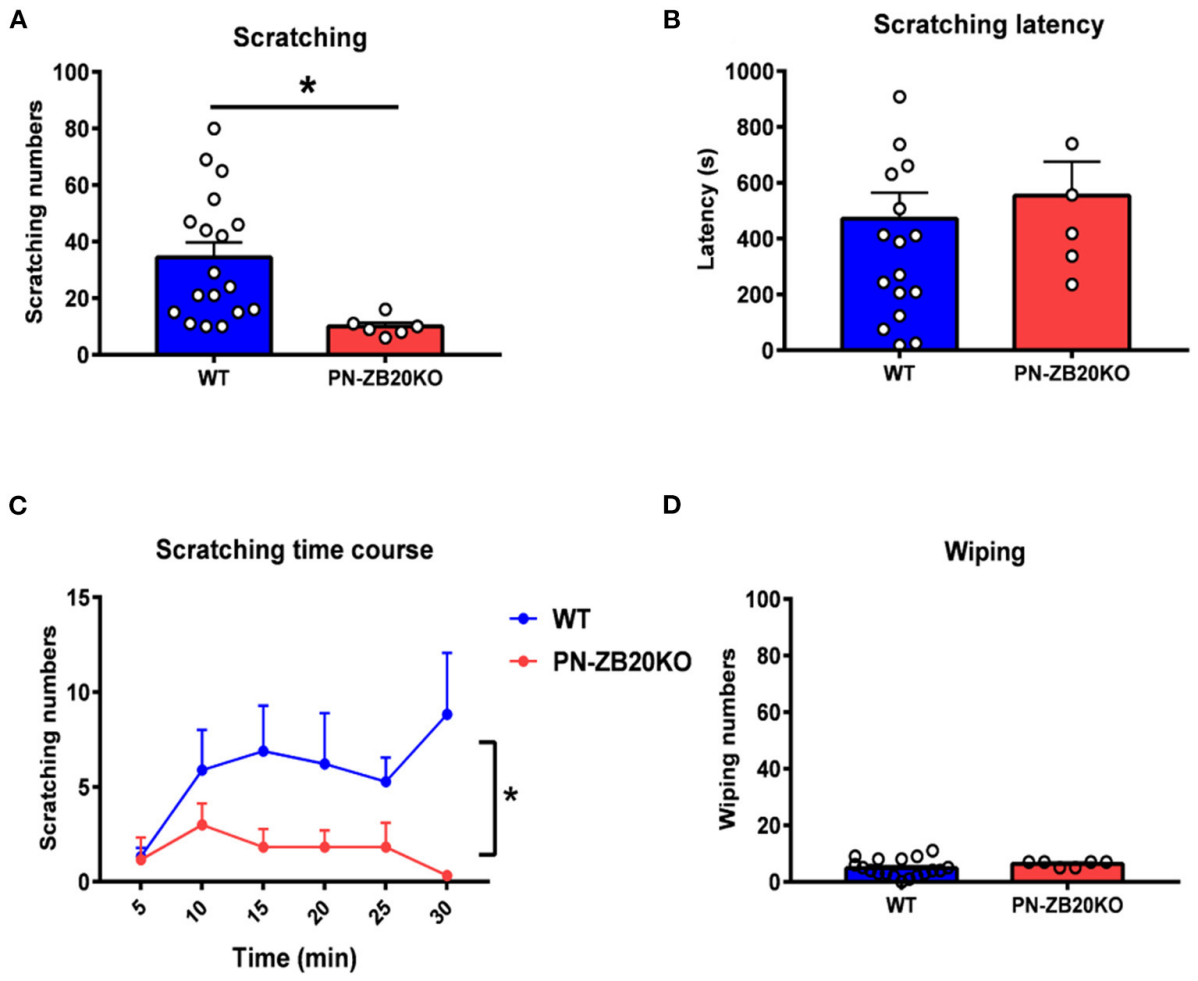
Histamine-induced itch was dramatically attenuated in PN-ZB20KO mice. **(A)** The scratching behavior induced by injection of histamine (50 μg in 10 μL PBS) into the mouse cheek was decreased significantly in PN-ZB20KO mice compared to control mice (*t*-test, *p < 0.05). **(B)** The latency to scratch following histamine injection did not change in PN-ZB20KO mice compared to control mice (*t*-test, *p* > 0.05). **(C)** The time course of scratching behavior induced by histamine administration. Two-way ANOVA, [F_(1, 22)_ = 6.989; *p* = 0.0148, *p < 0.05]. **(D)** The wiping induced by histamine was maintained at a low level and did not change after histamine administration (*t*-test, *p* > 0.05). *N* = 6–18 for each group.

**Figure 2:**
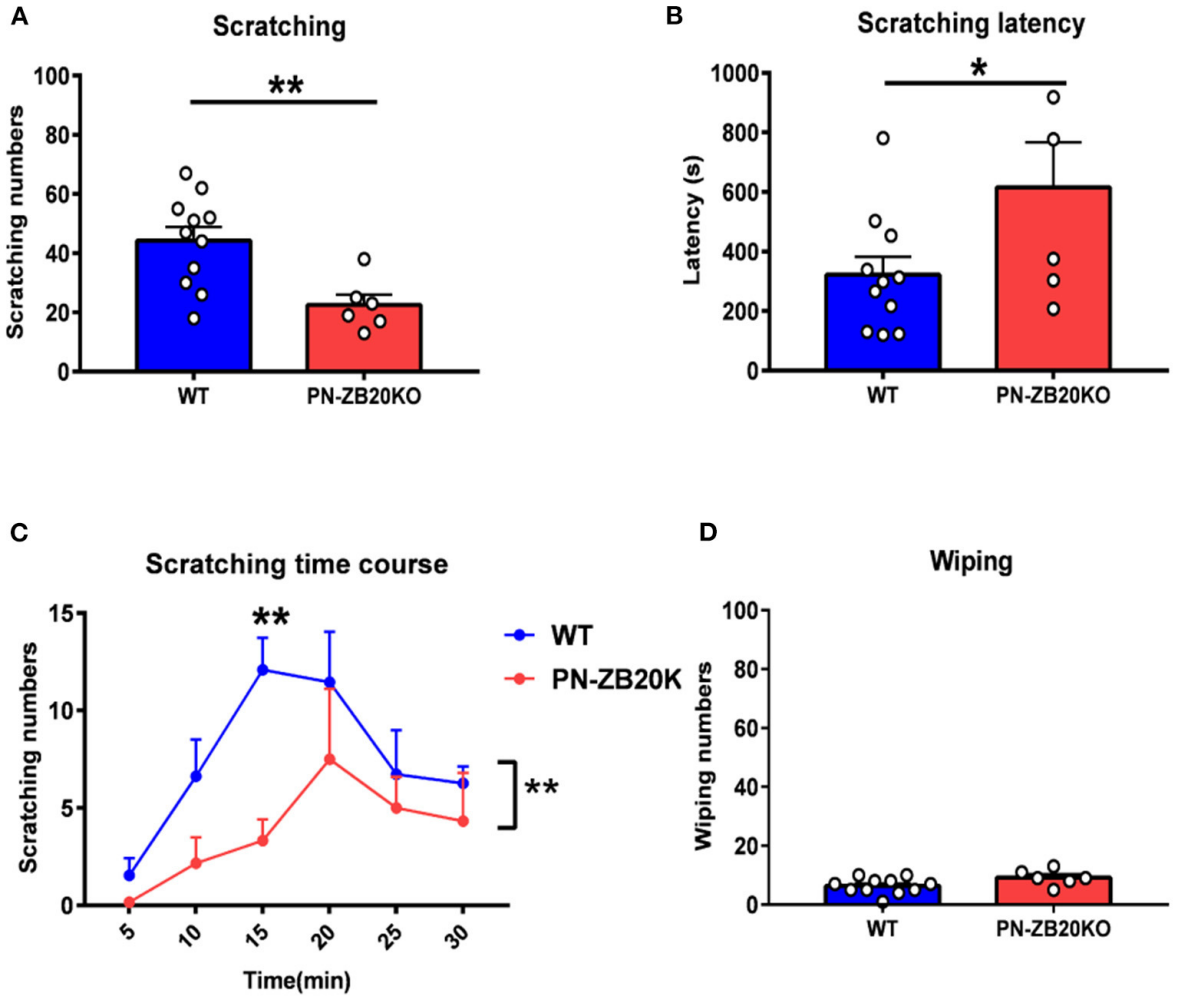
CQ-induced itch was dramatically attenuated in PN-ZB20KO mice. **(A)** The scratching behavior induced by injection of CQ (40 μg in 10 μL PBS) into the mouse cheek was decreased significantly in PN-ZB20KO mice compared to control mice (*t*-test, **p < 0.01). **(B)** The latency to scratch following CQ injection significantly increased in PN-ZB20KO mice compared to control mice (*t*-test, *p < 0.05). **(C)** The time course of scratching behavior induced by CQ administration. Two-way ANOVA [F_(1, 15)_ = 10.27; *p* = 0.0059, **p < 0.01]. **(D)** The wiping induced by CQ was maintained at a low level and did not change after histamine administration (*t*-test, *p* > 0.05). *N* = 6–11 for each group.

**Figure 3:**
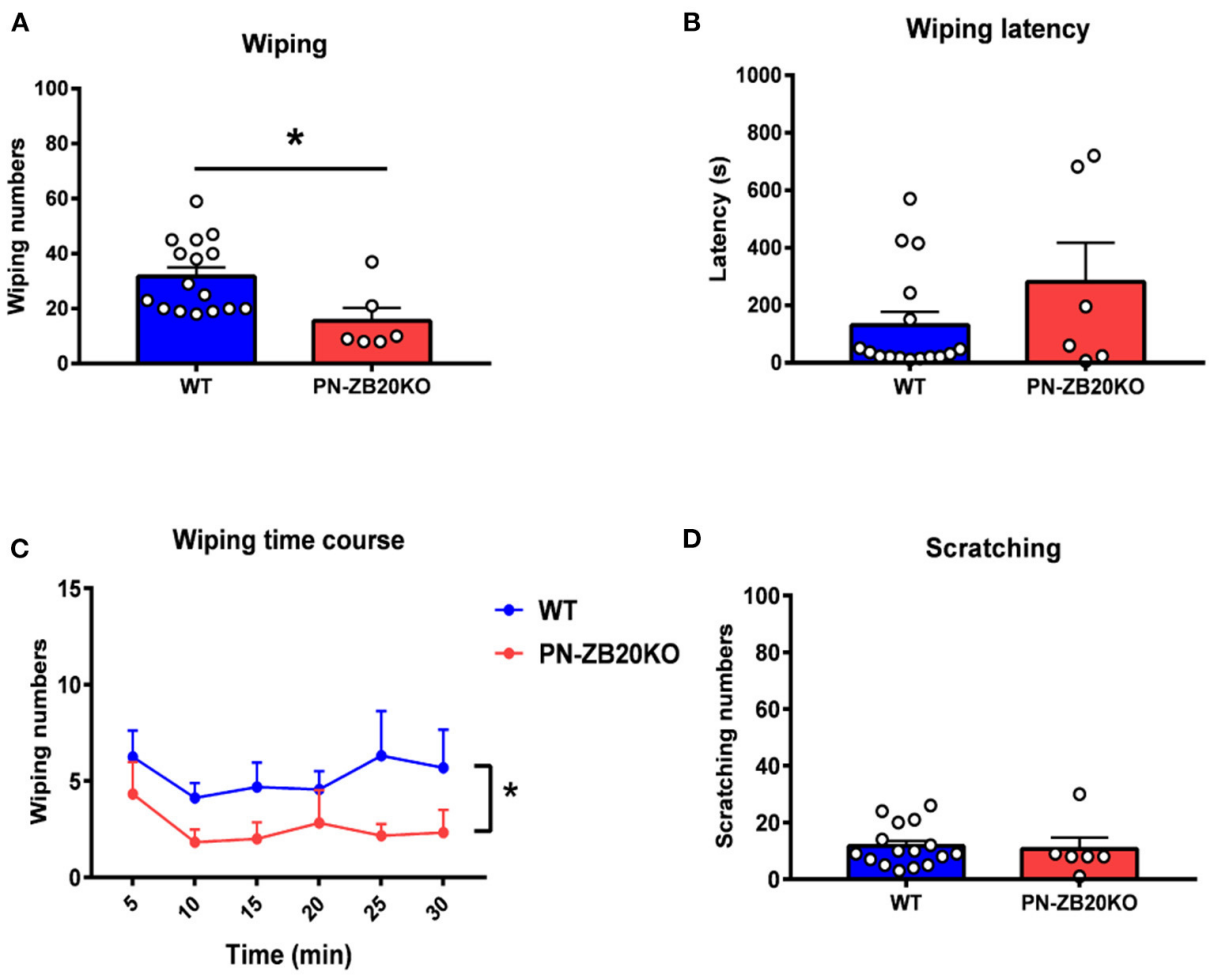
Capsaicin-induced wiping was dramatically attenuated in PN-ZB20KO mice. **(A)** The wiping behavior induced by injection of capsaicin (10 μg in 10 μL of solution) into the mouse cheek was decreased dramatically in PN-ZB20KO mice compared to control mice (*t*-test, *p < 0.05). **(B)** The latency to wiping following capsaicin injection did not change in PN-ZB20KO mice compared to control mice (*t*-test, *p* > 0.05). **(C)** The time course of wiping induced by capsaicin administration. Two-way ANOVA [*F*_(1, 20)_ = 6.958; *p* = 0.0158, *p < 0.05]. **(D)** The scratching induced by capsaicin was maintained at a low level and did not change after capsaicin administration. *N* = 6–16 for each group.

To examine whether ZBTB20 in primary neurons is involved in itch transmission, we first established a mouse cheek model to measure histamine- or CQ-induced itch behavior, which represent histamine- and non-histamine-dependent itch, respectively. After the pruritogens were injected into the cheek of each mouse, the total number of scratches and wipes every 5 min for 30 min was calculated. The latency to scratching or wiping behavior following chemical injection was also recorded. The results showed that scratching behaviors induced by histamine (5 μg/μL, 10 μL) (Figures 1A,C) and CQ (4 μg/μL, 10 μL) (Figures 2A,C) were robustly inhibited in PN-ZB20KO mice compared with WT mice. However, little forelimb wiping was observed in PN-ZB20KO mice, and there were no differences in this behavior between the two groups (Figures 1D, 2D). Furthermore, the latency to scratch following CQ injection was increased significantly in PN-ZB20KO mice compared with WT mice (Figure 2B), further indicating the attenuation of CQ-induced itch. The results above suggest that ZBTB20 in primary sensory neurons plays an important role in mediating histamine- and non-histamine-induced itch. In addition, the number of forelimb wipes induced by capsaicin was significantly reduced in PN-ZB20KO mice compared to WT mice (Figure 3), which is in line with a previous report (5) and further verifies the function of ZBTB20 in modulating inflammatory pain.

### ZBTB20 Is Expressed in Small Peptidergic Neurons and Colocalizes With TRP Channels in the TG

**Figure 4:**
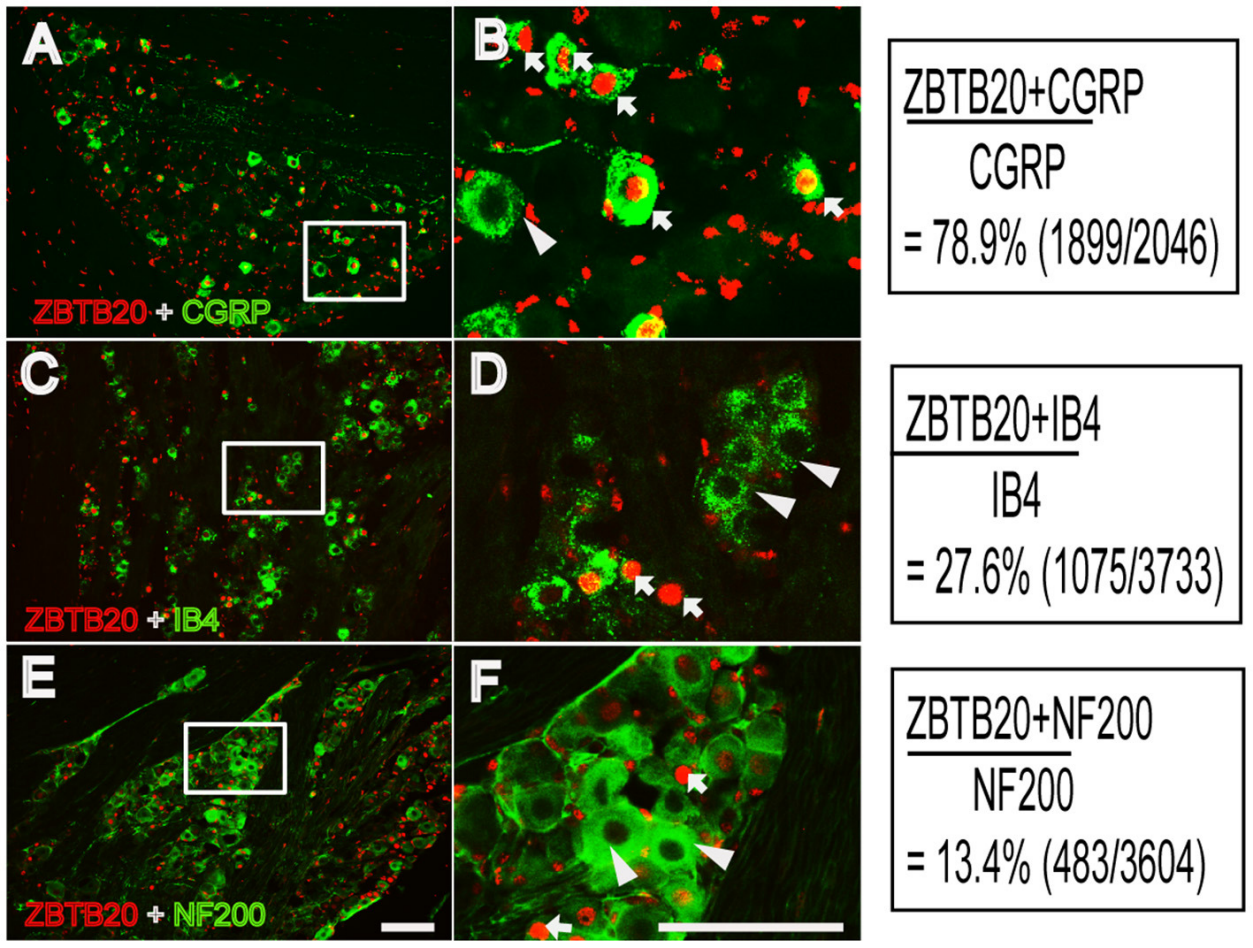
Colocalization of ZBTB20 with CGRP, IB4, and NF200 in the trigeminal ganglia. **(A,B)** Colocalization of ZBTB20 with CGPR. **(C,D)** Colocalization of ZBTB20 with IB4. **(E,F)** Colocalization of ZBTB20 with NF200. **Figures B,D,F** are high magnifications of the boxes in **Figures A,C,E**. The arrowhead indicates the colocalization of ZBTB20 with CGRP, IB4, and NF200 in **Figures B,D,F**. The arrow indicates the expression of CGRP, IB4, and NF200 without colocalization with ZBTB20 in **Figures B,D,F**. *N* = 3; bar =50 μm.

**Figure 5:**
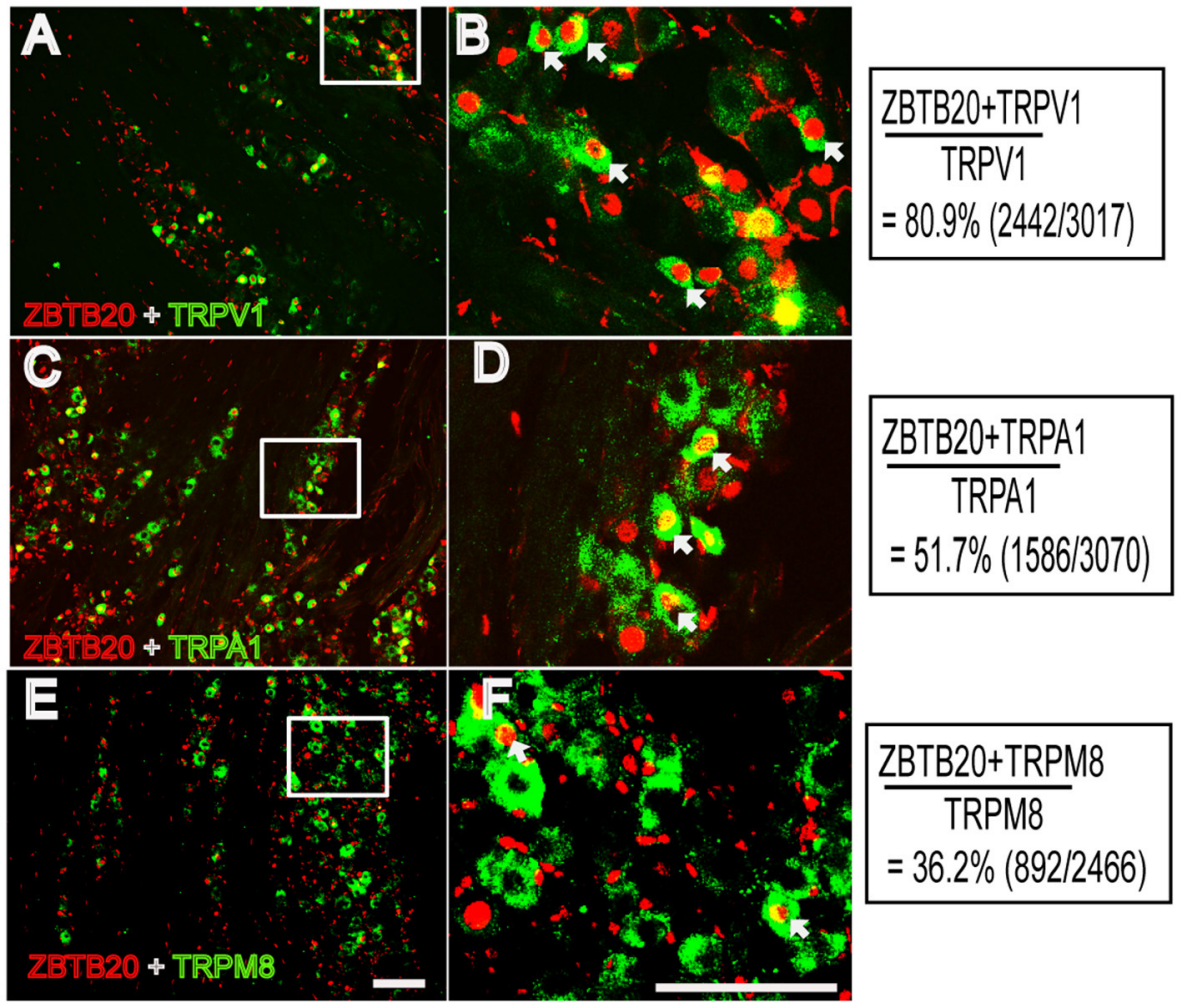
Colocalization of ZBTB20 with TRPV1, TPRA1, and TRPM8 in the trigeminal ganglia **(A,B)** Colocalization of ZBTB20 with TRPV1. **(C,D)** Colocalization of ZBTB20 with TRPA1. **(E,F)** Colocalization of ZBTB20 with TRPM8. **Figures B,D,F** are high magnifications of the boxes in **Figures A,C,E**. The arrowhead indicates the colocalization of ZBTB20 with TRPV1, TRPA1, and TRPM8 in **Figures B,D,F**. *N* = 3; bar =50 μm.

Previous studies have shown that ZBTB20 is colocalized with peripherin and exists in 80% of nav1.8-positive neurons, indicating that ZBTB20 mainly exists in small neurons in the DRG (5). However, the cellular distribution of ZBTB20 in primary sensory neurons, especially the percentage of ZBTB20 expressed in different TG neurons, has not been demonstrated. We detected the expression of ZBTB20 in the TG by double immunofluorescence, and the results showed that ZBTB20 was expressed in 78.9% of CGRP^+^ neurons (Figures 4A,B), 27.6% of IB4 ^+^ (Figures 4C,D) and 13.4% of NF200^+^ neurons (Figures 4E,F). We further investigated the colocalization of ZBTB20 with TRP channels and found that ZBTB20 was expressed in 80.9% of TRPV1^+^, 51.7% of TRPA1^+^ and 36.2% of TRPM8^+^ neurons in the TG (Figures 5A–F). These results suggested that ZBTB20 is mainly distributed in small peptidergic neurons and coexists with the majority of TRPV1 and TRPA1, implicating ZBTB20 in the modulation of both pain and itch.

### Pruritogens Increase the mRNA Expression of TRPV1 and TRPA1 in the TG

**Figure 6:**
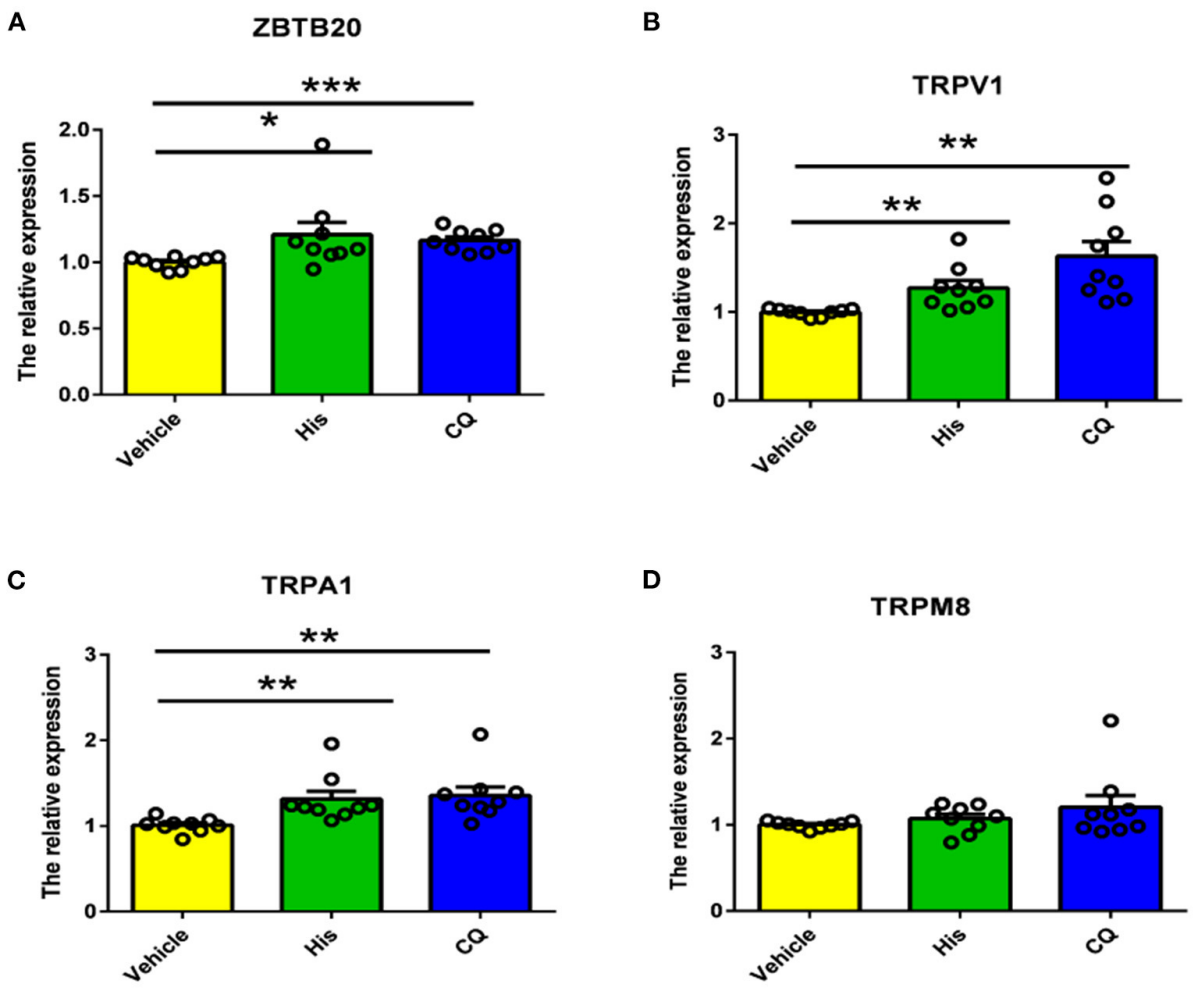
Histamine and CQ increased the mRNA expression of ZBTB20, TRPV1, and TRPA1 but not TRPM8 in the trigeminal ganglia. **(A)** The mRNA expression of ZBTB20 in the TG was increased after histamine and CQ injection into the cheek (*t*-test, *p < 0.05, ***p < 0.001 vs. the vehicle group). **(B)** The mRNA expression of TRPV1 in the TG was increased after histamine and CQ injection into the cheek (*t*-test, **p < 0.01 vs. the vehicle group). **(C)** The mRNA expression of TRPA1 in the TG was increased after histamine and CQ injection into the cheek (*t*-test, **p < 0.01 vs. the vehicle group). **(D)** The mRNA expression of TRPM8 in the TG was not increased after histamine and CQ injection into the cheek (*t*-test, *p* > 0.05 vs. the vehicle group). *N* = 9 for each group.

Because TRP channels are very important for itch transduction in primary neurons, we next measured the TRP channel mRNA expression induced by histamine and CQ. The results showed that the mRNA expression of TRPV1 and TRPA1 but not TRPM8 in the TG was upregulated significantly by cheek injection of histamine and CQ (Figures 6B–D). Moreover, the mRNA expression of ZBTB20 was also upregulated by cheek injection of the two types of pruritogens (Figure 6A). Our results indicated that ZBTB20 may mediate pruritus by regulating TRPV1 and TRPA1.

### TRPV1 and TRPA1 KO Mice Exhibit Attenuation of Itch Induced by Histamine and CQ

**Figure 7:**
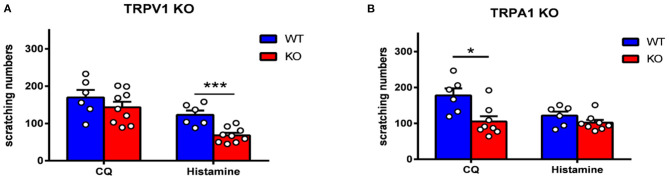
Itch behavior was attenuated in TRPV1 KO and TRPA1 KO mice. **(A)** Histamine-induced scratching was reduced dramatically in TRPV1 KO mice (*t*-test, ***p < 0.001 vs. the WT group). **(B)** CQ-induced scratching was reduced dramatically in TRPA1 KO mice (*t*-test, *p < 0.05 vs. the WT group). *N* = 6-9 for each group.

To further verify the role of TRPV1 and TRPA1 in itch sensation, TRPV1 and TRPA1 KO mice were used. We found that scratching induced by histamine was inhibited in TRPV1 KO mice compared to WT mice (Figure 7A), while scratching induced by CQ was attenuated in TRPA1 KO mice compared with WT mice (Figure 7B). The data are in line with a previous study (11, 18, 30, 31) and further suggest that TRPV1 and TRPA1 modulate histamine- and non-histamine-dependent acute itch.

### Silencing ZBTB20 in TG Suppressed Histamine-Dependent and Non-histamine-Dependent Itch

**Figure 8:**
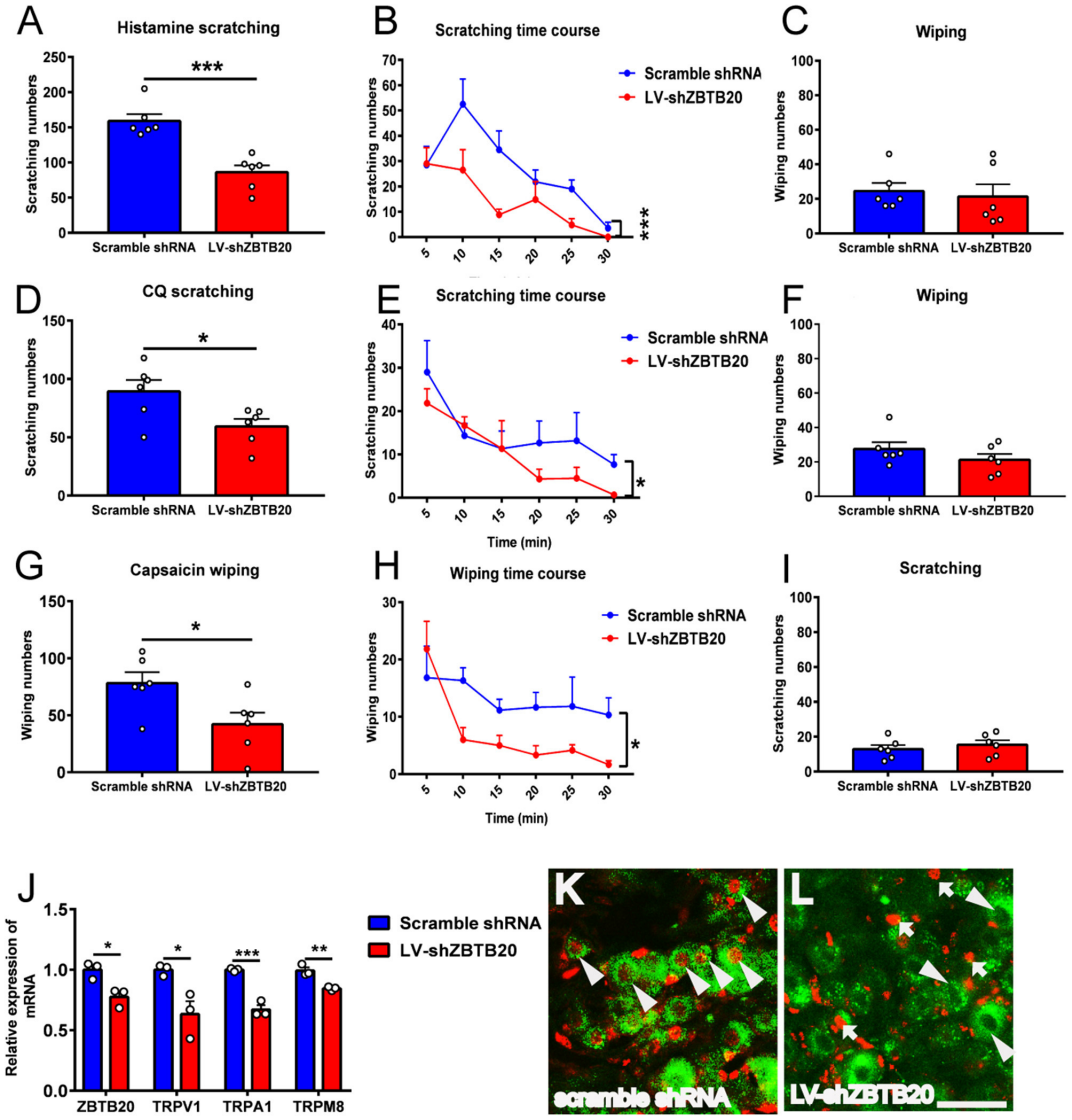
The itch and pain behavior induced by histamine, CQ or capsaicin after silencing endogenous ZBTB20 in the TG. **(A)** The scratching induced by histamine (50 μg in 10 μL PBS) was decreased dramatically in LV-shZBTB20 mice compared to scramble shRNA mice (*t*-test, ***p < 0.001). **(B)** The time course of scratching induced by histamine in LV-shZBTB20 and scramble shRNA mice [F_(1, 10)_ = 29.06; *p* = 0.0003, ***p < 0.001]. **(C)** The wiping induced by histamine was maintained at a low level and did not change after histamine injection (*t*-test, *p* > 0.05). **(D)** The scratching induced by CQ (4 μg in 10 μL PBS) was decreased dramatically in LV-shZBTB20 mice compared to scramble shRNA mice (*t*-test, *p < 0.05). **(E)** The time course of scratching induced by CQ LV-shZBTB20 and scramble shRNA mice [*F*_(1, 15)_ = 10.27; *p* = 0.0059, *p < 0.05]. **(F)** The wiping induced by CQ was maintained at a low level and did not change after CQ treatment (*t*-test, *p* > 0.05). **(G)** The wiping induced by capsaicin (10 μg in 10 μL of solution) was decreased dramatically in LV-shZBTB20 mice compared to scramble shRNA mice (*t*-test, *p < 0.05). **(H)** The time course of wiping induced by capsaicin in LV-shZBTB20 and scramble shRNA mice [*F*_(1, 10)_ = 6.548; *p* = 0.0284, *p < 0.05]. **(I)** The scratching induced by capsaicin was maintained at a low level and did not change after capsaicin treatment. *N* = 6 for each group. **(J)** The mRNA expression of ZBTB20, TRPV1, TRPA1, and TRPM8 in the TG was decreased significantly in LV-shZBTB20 mice compared to scramble shRNA mice (*t*-test, *p < 0.05, **p < 0.01, ***p < 0.001). *N* = 3 for each group. **(K,L)** Example showing immunofluorescent staining of ZBTB20 with scramble shRNA and LV-shZBTB20 in TG neurons. The arrowhead indicates colocalization of ZBTB20 (red) with scramble shRNA (green) **(K)**. The arrow indicates ZBTB20 (red), and the arrowhead indicates LV-shZBTB20 (green) **(L)**. *N* = 3; bar =50 μm.

To further verify the effect of ZBTB20 on itch behavior, we silenced endogenous ZBTB20 expression in primary mouse TG neurons using recombinant lentivirus expressing a short hairpin RNA against ZBTB20 (LV-shZBTB20) or a scramble shRNA as a mock control (29). The scratching numbers induced by histamine and CQ were dramatically attenuated by knocking down ZBTB20 in the TG (Figures 8A–F). In addition, the wiping numbers induced by capsaicin were also significantly attenuated after ZBTB20 RNA interference in the TG (Figure 8). The mRNA levels of ZBTB20, TRPV1, TRPA1, and TRPM8 were significantly decreased in the LV-shZBTB20 group compared with the scrambled shRNA control group (Figure 8J). After the behavior test, we also verified the knockdown effect of LV-shZBTB20 on ZBTB20 expression in the TG by immunofluorescence (Figures 8K,L).

## Discussion

In the present study, we used PN-ZB20KO mice and RNA interference to assess the function of ZBTB20 in the progression of itch. Our results showed that ZBTB20 in primary sensory neurons was involved in both histamine- and non-histamine-dependent itch and that the effect was likely mediated by TRPA1 and TRPV1 channels.

ZBTB20 is highly expressed in the nervous system and is essential for postnatal survival in mice (1). As previously described, the ZBTB20 gene is deleted specifically in nociceptors at E14 using Nav1.8-Cre, but deletion of this gene does not affect the formation, survival or diversification of nociceptors (5). It is well-known that pain and itch are transmitted by nociceptive primary sensory neurons. Hence, these PN-ZB20KO mice are suitable for the study of pain as well as itch. Given that ZBTB20 affects pain by regulating TRP channels in primary sensory neurons, it is worth investigating whether ZBTB20 regulates itch.

Pain and itch are distinguished by unique behavioral responses. While pain leads to withdrawal reflexes and other types of avoidance behavior, itch induces the urge to scratch. In the standard rodent model of itch, pruritogens are applied to the nape of the neck, and scratches with the hindpaw are evaluated and considered itch-responsive behavior (32, 33). However, mice also scratch with the hindpaw when capsaicin, which induces pain sensation, is injected into the nape of the neck. It was discovered that when the agents are injected into the cheek, mice scratch the injection site with their hind limbs in response to histamine (itch) and wipe with their forelimbs in response to capsaicin (pain) (19). Therefore, since 2008, the mouse cheek model reported by Shimada and LaMotte has been widely used to differentiate itch and pain behaviors. In our study, scratch behavior was largely attenuated in PN-ZB20KO mice compared to WT mice when histamine and CQ were administered to the cheek, indicating that ZBTB20 in primary sensory neurons modulates both histamine- and non-histamine-dependent itch sensation. In addition, we found that wiping behavior induced by capsaicin was dramatically decreased in PN-ZB20KO mice compared to WT mice, which is in line with our previous report (5), showing that ZBTB20 is involved in inflammatory pain; these data further confirm the function of ZBTB20 in modulating both pain and itch.

Nociceptive primary sensory neurons are located in the DRG and TG, which are homologs of each other and transmit nociception from the body and craniofacial neurons, respectively. The nociceptive neurons in the DRG and TG can be chemically divided into two subsets: peptidergic and non-peptidergic neurons. Peptidergic neurons synthesize neuropeptides such as calcitonin gene-related peptide (CGRP) and substance P (SP) and respond to nerve growth factor. Non-peptidergic neurons, which express the c-Ret neurotrophin receptor, are capable of binding isolectin IB4 and responding to glial-derived neurotrophic factors (34, 35). Because we administered chemicals to the cheek in this study, ZBTB20 expression in the TG was measured. We therefore used CGRP and IB4 to label these two subsets of neurons in the TG and found that ZBTB20 was expressed in most CGRP ^+^ small peptidergic neurons and a few IB4^+^ small non-peptidergic neurons and NF200^+^ large neurons. This result further suggests that ZBTB20 is involved in regulating TRP channels, since TRPV1 is expressed in a population of unmyelinated neurons that express the neuropeptide CGRP within rodent sensory ganglia (7). Although ZBTB20 is expressed by precursor cells for all neuronal types (36), it becomes more restricted in the majority of neurons expressing TRPV1, TRPA1, and TPRM8 in the DRG (5). To further investigate the colocalization of ZBTB20 with TRP channels in TG neurons, we performed double immunostaining for ZBTB20 and TRP channels and found that ZBTB20 was expressed in most TRPV1^+^ (80.9%) and TRPA1^+^ (51.7%) neurons and to a lesser extent in TRPM8^+^ (36.2%) neurons in the TG, suggesting that there may be differences in neuron mechanisms in the DRG and TG where primary sensory neurons are located. Nevertheless, our results confirmed a previous report and provided detailed information regarding the expression of ZBTB20 in the TG.

TRP channels are molecular sensors for mechanical, chemical, and thermal changes. Recently, growing evidence has indicated that TRP channels also play an important role in itch signaling (13), and different TRP channels are required for different types of itch. For example, TRPV1 mediates histamine-induced itch by coupling with histamine H1R and H4R (32, 37). TRPA1 is involved in non-histamine-dependent itch induced by CQ and BAM8-22. TRPV1 or TRPA1 KO mice exhibit less histamime- or CQ-evoked scratching behavior (18). In addition, some pruritogens, such as lysophosphatidic acid (LPA), squaric acid dibutylester (SADBE) and IL31, induce itch that is mediated by both TRPV1 and TRPA1 (38–40). It has been demonstrated that mice with TRPV1 exclusively expressed in MrgprA3^+^ neurons exhibit only itch and not pain behavior in response to capsaicin (22), indicating that there are two subpopulations of TRPV1 neurons in the primary sensory ganglia that distinctly mediate itch and pain. However, whether ZBTB20 regulates itch-related TRP channels has not been reported. Our data demonstrated that both histamine and CQ increase the mRNA expression of ZBTB20, TRPV1, and TRPA1 in the TG, providing evidence that ZBTB20 probably modulates TRP channels in itch-specific neurons. In contrast to TRPV1 and TRPA1, TRPM8 inhibits itch and is required for cooling and menthol-mediated itch inhibition (41). Although previous results have shown that ZBTB20 affects pain behavior by regulating TRPA1, TRPV1, and TRPM8, in our study, TRPM8 expression was not altered after pruritogen administration, further indicating that pain and itch are two different modalities that have distinct molecular signaling pathways.

In summary, our results demonstrated that ZBTB20 acts as a critical regulator of pruritus in primary sensory neurons, which could through TRPA1 and TRPV1 channels. Our study will help to unravel the cellular and molecular bases of itch sensation.

## Data Availability Statement

The original contributions presented in the study are included in the article/supplementary material, further inquiries can be directed to the corresponding author/s.

## Ethics Statement

The animal study was reviewed and approved by Animal Study Committee of Tongji University School of Medicine Shanghai, China.

## Author Contributions

XJ and M-HD performed the behavioral experiments and RT-PCR. XJ and A-JR performed RNA interference experiments. T-TW participated in the behavior experiments. T-TW and A-JR performed the immunohistochemistry experiments. WZ and LZ designed the experiments and wrote the manuscript. All authors contributed to the article and approved the submitted version.

## Conflict of Interest

The authors declare that the research was conducted in the absence of any commercial or financial relationships that could be construed as a potential conflict of interest.
